# iTRAQ Proteomic Analysis Reveals That Metabolic Pathways Involving Energy Metabolism Are Affected by Tea Tree Oil in *Botrytis cinerea*

**DOI:** 10.3389/fmicb.2017.01989

**Published:** 2017-10-12

**Authors:** Jiayu Xu, Xingfeng Shao, Yingying Wei, Feng Xu, Hongfei Wang

**Affiliations:** Department of Food Science and Engineering, Ningbo University, Ningbo, China

**Keywords:** iTRAQ, proteomics, essential oil, *Botrytis cinerea*, antifungal

## Abstract

Tea tree oil (TTO) is a volatile essential oil obtained from the leaves of the Australian tree *Melaleuca alternifolia* by vapor distillation. Previously, we demonstrated that TTO has a strong inhibitory effect on *Botrytis cinerea*. This study investigates the underlying antifungal mechanisms at the molecular level. A proteomics approach using isobaric tags for relative and absolute quantification (iTRAQ) was adopted to investigate the effects of TTO on *B. cinerea*. A total of 718 differentially expression proteins (DEPs) were identified in TTO-treated samples, 17 were markedly up-regulated and 701 were significantly down-regulated. Among the 718 DEPs, 562 were annotated and classified into 30 functional groups by GO (gene ontology) analysis. KEGG (Kyoto Encyclopedia of Genes and Genomes) enrichment analysis linked 562 DEPs to 133 different biochemical pathways, involving glycolysis, the tricarboxylic acid cycle (TCA cycle), and purine metabolism. Additional experiments indicated that TTO destroys cell membranes and decreases the activities of three enzymes related to the TCA cycle. Our results suggest that TTO treatment inhibits glycolysis, disrupts the TCA cycle, and induces mitochondrial dysfunction, thereby disrupting energy metabolism. This study provides new insights into the mechanisms underlying the antifungal activity of essential oils.

## Introduction

*Botrytis cinerea*, one of the most destructive fungal pathogens, causing gray mold rot in a wide range of fresh fruits and vegetables. The resulting reduction in shelf life is responsible for enormous economic losses in the produce industry. Although chemical fungicides are widely used to control the incidence of the disease, this practice potentially introduces harmful substances into the food chain, and also selects for *B. cinerea* strains with increased drug resistance (Brul and Coote, 1999; Leroux et al., 2002). These limitations provide a strong stimulus to explore safer and more effective antifungal agents. Essential oils are promising natural substitutes that offer disease control by inhibiting pathogen growth (Prakash et al., 2012). For example, the essential oils of *Angelica archangelica* L. (*Apiaceae*) roots and *Solidago canadensis* L. have been characterized and tested *in vitro* as antifungal agents against *B. cinerea* (Fraternale et al., 2014; Liu et al., 2016). Lemongrass essential oil significantly reduces the incidence of *B. cinerea* and prolongs the shelf-life and sensory properties of frozen mussels and vegetables (Abdulazeez et al., 2016). Essential oils of aromatic plants, which belong to the Lamiacea family such as origanum (*Origanum syriacum* L. var. *bevanii*), lavender (*Lavandula stoechas* L. var. *stoechas*) and rosemary (*Rosmarinus officinalis* L.), have been reported to cause considerable morphological degenerations of the fungal hyphae of *B. cinerea* and suppress *in vivo* disease development on tomato against *B. cinerea* (Soylu et al., 2010).

Tea tree oil (TTO) is a volatile natural plant essential oil obtained from the leaves of the Australian tree *Melaleuca alternifolia* by vapor distillation (Homer et al., 2000). The oil exhibits a broad spectrum of antimicrobial activities against a variety of bacteria, fungi, and virus (Carson et al., 2006; Miao et al., 2016). Growth and metabolic activity of *Escherichia coli* and *Candida albicans* are inhibited after treatment with TTO (Gustafson et al., 1998; Bona et al., 2016). Our previous studies showed that TTO treatment effectively inhibits spore germination and mycelial growth of *B. cinerea*, modifies its morphology and cellular ultrastructure, and controls gray mold on strawberry and cherry fruits (Shao et al., 2013a; Li et al., 2017a). TTO's antifungal mechanism in *B. cinerea* involves the loss of membrane integrity and the subsequent release of intracellular compounds, probably due in part to changes in membrane fatty acid and ergosterol composition (Shao et al., 2013b; Li et al., 2017a). TTO also causes mitochondrial damage in *B. cinerea*, disrupting the tricarboxylic acid (TCA) cycle and leading to the accumulation of reactive oxygen species (ROS) (Li et al., 2017b). Metabolomic analysis by quadrupole time-of-flight mass spectrometer was consistent with these results (Xu et al., 2017). However, the molecular mechanisms underlying the effects of TTO against *B. cinerea* have not yet been associated with specific proteins.

Proteomics can be used to study the changes in protein levels under stress conditions in great detail (Franco et al., 2013), and has been applied to investigate the mode of action of the antimicrobial agent apidaecin IB against membrane proteins in *E. coli* cells (Zhou and Chen, 2011). Other studies have revealed that proteins related to energy and DNA metabolism, and amino acid biosynthesis are down-regulated in *E. coli* JK-17 in the presence of rose flower extract (Cho and Oh, 2011). *Syzygium aromaticum* essential oil perturbs the expression of virulence-related genes involved in the synthesis of serine protease, flagella, and lipopolysaccharide in *Campylobacter jejuni* (Kovács et al., 2016). In this study, we conducted a proteomics analysis using isobaric tags for relative and absolute quantification (iTRAQ) to study *B. cinerea* to identify proteins and potential mechanisms underlying the antifungal activity of TTO.

## Materials and methods

### *B. cinerea* growth and exposure to TTO

Highly virulent *B. cinerea* (ACCC 36028) was purchased from the Agricultural Culture Collection of China and grown at 25°C on potato dextrose agar (PDA, containing 1 L potato liquid, 20 g/L glucose, and 15 g/L agar) before use. TTO was purchased from Fuzhou Merlot Lotus Biological Technology Company (Fujian Province, China). The primary components of TTO are terpinen-4-ol (37.11%), γ-terpinene (20.65%), α-terpinene (10.05%), 1, 8-cineole (4.97%), terpinolene (3.55%), ρ-cymene (2.14%), and α-terpineol (3.82%), as specified by the supplier. *B. cinerea* cultures were maintained on PDA at 25°C for 3 days. Spore suspensions were harvested by adding 10 mL sterile 0.9% NaCl solution to each petri dish and then gently scraping the mycelial surface three times with a sterile L-shaped spreader to free the spores. The spore suspension was adjusted using a hemocytometer to 5 × 10^6^ spores/mL. One milliliter suspension was inoculated into 250 mL flasks containing 150 mL sterile potato dextrose broth medium and cultured at 25°C on a rotary shaker at 150 revolutions per minute for 3 days. Before mycelia were harvested, TTO was added to the medium to a final concentration of 5 mL/L, and cultures incubated for another 2 h (Xu et al., 2017). Mycelia were collected and rinsed three times with 0.1 M phosphate buffered saline (PBS) (pH 7.4). Samples were stored at −80°C. Cultures without TTO were used as a control. Three samples were prepared in parallel for each condition.

### Protein extraction

Approximately 200 mg of frozen mixed mycelium from control or TTO treated cultures was ground into powder in liquid nitrogen and suspended in 25 mL 10% (v/v) trichloroacetic acid in acetone containing 65 mM dithiothreitol (DTT). The suspension was vortexed and incubated at −20°C for 2 h, centrifuged at 12,000 × g for 45 min at 4°C, and the supernatant discarded. The precipitate was rinsed three times with chilled acetone. The pellet was vacuum dried and dissolved in lysis buffer (4% SDS, 100 mM Tris-HCl, 100 mM DTT, pH 8.0). After incubation for 5 min in boiling water, the suspension was sonicated on ice at 50 W for 5 min. The crude extract was incubated in boiling water again for 5 min, and clarified by centrifugation at 14,000 × g for 40 min at 20°C. To digest protein in the supernatant, 200 μL UA buffer (8 M urea, 150 mM Tris-HCl, pH 8.5) was added and the mixture was centrifuged at 14,000 × g for 30 min at room temperature. This step was repeated three times. Subsequently, 100 μL 50 mM iodoacetamide (IAM) was added, the samples were incubated for 30 min in darkness, and then centrifuged at 14,000 × g for 30 min at room temperature. The precipitate was resuspended in 100 μL UA buffer and samples were centrifuged at 14,000 × g for 30 min at room temperature. 100 μL dissolution buffer was added, followed by centrifugation at 14,000 × g for 30 min at room temperature. This step was repeated three times. The supernatant was removed, the pellet was dissolved in 40 μL trypsin buffer, incubated at 37°C for 18 h, and clarified by centrifugation at 14,000 × g for 30 min at room temperature. Finally, 40 μL 25 mM dissolution buffer was added and samples were centrifuged at 14,000 × g for 30 min at room temperature. The supernatant was transferred to a new tube and quantified with the Bradford assay using BSA as the standard, and SDS-PAGE was performed to verify protein quality.

### iTRAQ labeling and strong cation exchange (SCX) fractionation

iTRAQ labeling was performed according to the manufacturer's instructions. Peptides were prepared using the 8-plex iTRAQ labeling kit (AB Sciex, CA, USA). Control replicates were labeled with reagents 113, 114, and 115, and the TTO treatment replicates were labeled with reagents 116, 117, and 118. The labeled peptide mixtures were pooled and dried by vacuum centrifugation.

The labeled peptide mixtures were dissolved in 3 mL buffer A (10 mM KH_2_PO_4_ in 25% acetonitrile, pH 3.0) and loaded onto a polysulfoethyl 4.6 × 100 mm column (5 μm, 200 Å, PolyLC, Inc., Maryland, USA). The peptides were eluted at a flow rate of 1 mL/min with a gradient of buffer A for 30 min, 5–70% buffer B (10 mM KH_2_PO_4_, 500 mM KCl in 25% acetonitrile, pH 3.0) for 65 min, and 70–100% buffer B for 80 min. The eluted peptides were pooled into 10 fractions, desalted on C18 cartridges (Sigma), and vacuum-dried.

### LC-MS/MS analysis

For nano LC–MS/MS analysis, 10 μL of supernatant from each fraction was injected into an Obitrap-Elite (ThermoFinnigan) equipped with an Easy nLC (Proxeon Biosystems, now Thermo Fisher Scientific). The mobile phase was a mixture of water containing 0.1% formic acid and acetonitrile with 0.1% formic acid isocratically delivered by a pump at a flowrate of 250 nL/min. The elution gradient was: 0–105 min, 0–50% B; 105–110 min, 50–100% B; 110–120 min, 100% B. The MS scanning range was 300–1,800 m/z, MS resolution was 70,000, the number of scans range was 1, and the dynamic exclusion time was 40 s. The MS/MS activation type was HCD, the isolation window was 2 m/z, the MS/MS resolution was 17,500, the normalized collision energy was 30 eV, and the underfill ratio was 0.1%.

### Analysis of differentially expression proteins

For protein quantitation, one protein was required to contain at least two unique peptides. The quantitative protein ratios were weighted and normalized by the median ratio in Mascot (http://www.matrixscience.com). When differences in protein expression between TTO-treated and control groups were >1.5-fold or <0.67-fold, with *p* < 0.05, the protein was considered to be differentially expressed.

### Bioinformatic analysis

Gene Ontology (GO) is a standardized gene function classification system that describes the properties of proteins using three attributes: biological process, molecular function, and cellular components (Ashburner et al., 2000). A GO analysis (http://www.geneontology.org) was conducted to assign functional annotations for differentially expression proteins (DEPs), and the Kyoto Encyclopedia of Genes and Genomes (KEGG) (http://www.genome.jp/kegg) was used to predict the primary metabolic and signal transduction pathways in which the identified DEPs are involved.

### Confocal laser scanning microscopy

To assess the effects of TTO on the cytoplasmic membranes of *B. cinerea*, confocal laser scanning microscopy (LSM 880, Carl Zeiss, Germany) was performed, using the fluorescent indicator propidium iodide (PI) (Sigma-Aldrich, USA) and a modified protocol (Lee and Kim, 2017). *B. cinerea* cells containing 4 × 10^6^ spores/ml were added to each glass tube and incubated with TTO (final concentration 5 mL/L) with shaking at 200 rpm at 25°C for 2 h. The cells were washed and resuspended in 0.5 mL PBS (pH 7.4), stained with PI (10 μM final concentration) for 30 min at room temperature in the dark, and then washed twice with PBS. Images were acquired using confocal laser scanning microscopy. The experiment was repeated three times.

### Measurement of enzyme activities related to TCA cycle

Using the protocol described above (see Protein Extraction), ground mycelium was suspended in PBS (pH 7.4) and centrifuged at 10,000 × g for 10 min at 4°C. Enzyme activities were measured in the supernatant for malate dehydrogenase (MDH), citrate synthase (CS), and oxoglutarate dehydrogenase (OGDH), using kits purchased from Nanjing Jiancheng Bioengineering Institute (Nanjing, Jiangsu, China), following the manufacturer's instructions. Protein concentration was determined using a method based on the (Bradford, 1976) assay. MDH activity was calculated as μmol of NAD reduced per minute per mg of protein (U/mg protein). One unit of CS activity was defined as the amount of enzyme that produces 1 μmol of citric acid per minute (U/mg protein). OGDH activity was defined as the amount of enzyme that produces 1 nmol of NADH per minute (U/mg protein). Measurements were performed at 595 nm using three replicates for each sample.

### Statistical analysis

All experiments were repeated three times. Mean values and standard deviations were calculated using Excel 2010 (Microsoft Inc., Seattle, WA, USA). Statistical analyses were performed using one-way ANOVA with SPSS Statistics 17.0 (SPSS Inc., Chicago, USA).

## Results

### Identification of *B. cinerea* proteins by iTRAQ

**Figure 1:**
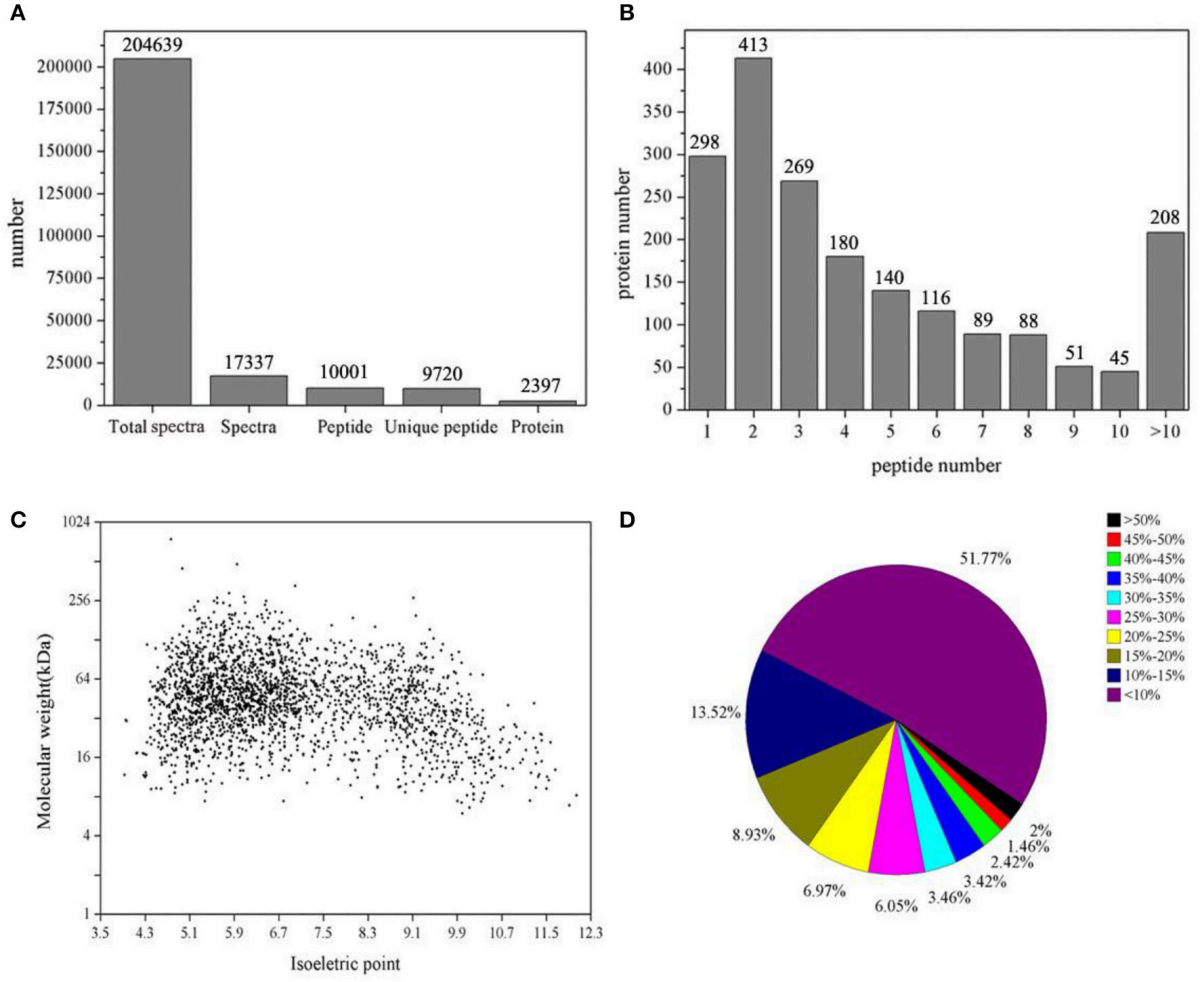
Summary of iTRAQ results. **(A)**, total spectra, matched spectra, matched peptides, unique peptides, and identified proteins. **(B)**, number of peptides associated with identified proteins. **(C)**, molecular weights vs. isoelectric points, as calculated from protein sequences. **(D)**, sequence coverage for identified proteins.

A total of 204,639 spectra were generated by iTRAQ proteomic analysis using control and TTO-treated *B. cinerea* and were analyzed using the Mascot search engine. As shown in Figure 1A, 17,337 spectra matched known spectra, comprising 10,001 peptides, 9,720 unique peptides, and 2,397 proteins from control and TTO-treated samples. The distribution of the number of peptides, predicted molecular weights, and isoelectric points, and peptide sequence coverage are shown in Figures 1B–D, respectively. Over 87% of the proteins were represented by at least two peptides. Molecular weights ranged from 20 to 200 kDa, and isoelectric points ranged from 5.0 and 7.0. Approximately 51% of identified proteins had more than 10% peptide sequence coverage.

### Identification of differentially expressed proteins using iTRAQ

The threshold for differential expression (TTO-treated vs. control) was a protein level difference >1.5 or < 0.67, with a *p* < 0.05. 718 differentially expressed proteins were identified in the TTO sample, of which 17 were up-regulated and 701 were down-regulated. Details for each protein are provided in Table 1.

**Table 1:** The main differentially expressed proteins in *B. cinerea* after treatment with TTO.

**Accession**	**Protein name**	**Score**	**Sequence coverage (%)**	**Fold^a^**	***p*-value**
gi|154691848	cytochrome c	96.3	37.9	0.328	0.007
gi|347441783	citrate synthase	133.1	8.0	1.819	0.028
gi|472236008	malate dehydrogenase protein	957.7	55.4	2.120	0.017
gi|472241505	oxoglutarate dehydrogenase protein	698.3	27.2	1.611	0.037
gi|347827327	pyruvate carboxylase	2, 263.6	38.7	1.751	0.027
gi|347833674	phosphoenolpyruvate carboxykinase	548.7	30.2	1.625	0.044
gi|347839725	succinyl-CoA ligase subunit alpha	420.3	24.3	1.612	0.040
gi|347826865	fructose-1,6-bisphosphatase	308.1	39.1	1.640	0.031
gi|154323902	enolase	2, 009.9	46.6	1.621	0.008
gi|472238209	glucose-6-phosphate isomerase protein	574.2	29.9	1.980	0.032
gi|472246374	phosphoglycerate mutase protein	54.3	2.6	1.576	0.021
gi|472240435	6-phosphofructokinase protein	539.9	28.1	1.775	0.022
gi|472237248	bisphosphoglycerate-independent phosphoglycerate mutase protein	823.0	44.1	2.164	0.018
gi|347841748	fructose-bisphosphate aldolase	1, 045.2	42.2	1.725	0.027
gi|536718572	phosphoglycerate kinase 1	587.5	40.2	1.723	0.040
gi|347833674	phospho-2-dehydro-3-deoxyheptonate aldolase	548.7	30.2	1.870	0.029
gi|347835540	phosphoglycerate mutase family protein	36.0	4.7	1.792	0.015
gi|472240974	6-phosphofructo-2-kinase fructose bisphosphatase protein	98.8	9.4	1.851	0.037
gi|347441437	inosine 5-monophosphate dehydrogenase	581.8	19.9	1.606	0.020
gi|347841600	adenine phosphoribosyltransferase	182.4	37.8	1.777	0.022
gi|347829189	adenosine kinase	465.9	31.3	1.956	0.016
gi|347441679	adenosylhomocysteinase	1, 287.4	61.7	1.881	0.027
gi|347837737	S-adenosylmethionine synthetase	423.1	30.1	2.004	0.008
gi|347831618	AMP deaminase 3	111.1	4.5	1.673	0.029
gi|347828730	adenylosuccinate synthetase	333.0	30.9	1.602	0.036
gi|347837737	S-adenosylmethionine synthetase	423.1	30.1	2.004	0.008
gi|347837845	adenylyl cyclase-associated protein	417.9	20.7	1.810	0.022
gi|472242224	guanyl-nucleotide exchange factor protein	65.4	1.5	1.674	0.004
gi|154691052	uracil phosphoribosyltransferase	90.6	9.4	1.796	0.046
gi|154697015	nucleoside diphosphate kinase	522.4	42.8	1.935	0.010
gi|347840376	UTP-glucose-1-phosphate uridylyltransferase	1, 333.6	45.7	1.623	0.038
gi|347832865	ribulose-phosphate 3-epimerase	38.6	7.9	2.204	0.031
gi|154300519	alcohol dehydrogenase protein	167.7	16.5	1.960	0.026
gi|347836330	alcohol dehydrogenase (NADP dependent)	281.1	24.4	2.019	0.020
gi|347441899	zinc-containing alcohol dehydrogenase	636.5	44.8	1.656	0.032
gi|347440923	aldehyde dehydrogenase	1, 070.9	48.0	1.865	0.021
gi|154703069	ATP synthase D chain, mitochondrial	252.1	26.4	1.924	0.050
gi|563298521	ATP synthase subunit e, mitochondrial	60.2	9.9	1.757	0.033
gi|347839842	ATP citrate lyase subunit	549.0	37.5	1.589	0.023
gi|154703371	vacuolar ATP synthase subunit E	93.3	12.7	2.382	0.013
gi|154692979	vacuolar ATP synthase subunit D	74.8	19.5	1.715	0.024
gi|347441643	vacuolar ATP synthase subunit H	307.6	22.3	1.761	0.028
gi|472245494	vacuolar ATP synthase catalytic subunit a protein	577.7	27.8	1.580	0.012
gi|347835157	v-type proton ATPase subunit B	274.1	17.6	2.041	0.019
gi|507414597	mitochondrial import protein 1	31.1	8.6	1.872	0.043
gi|472243251	mitochondrial pyruvate dehydrogenase kinase protein	61.4	3.4	2.632	0.009
gi|229891130	amino-acid acetyltransferase, mitochondrial	44.2	2.1	2.115	0.022
gi|3282211	isocitrate lyase 1, partial	27.8	2.5	1.874	0.029
gi|347832197	malate synthase	46.4	5.7	1.875	0.048
gi|347840647	acetyl-CoA carboxylase	2, 370.7	33.8	1.622	0.039
gi|347842358	acetyl-CoA acetyltransferase	449.4	46.3	1.982	0.018
gi|347841050	fatty acid synthase	1, 414.5	25.2	1.693	0.042
gi|472245418	fatty acid synthase beta subunit dehydratase protein	1, 668.6	24.8	1.567	0.045
gi|347841364	NADP-specific glutamate dehydrogenase	1, 138.8	46.9	1.840	0.021
gi|347827914	homocitrate synthase	454.5	39.0	1.501	0.031
gi|347837008	homoserine kinase	190.1	28.7	1.920	0.042
gi|347836521	GABA transaminase	483.9	27.7	1.544	0.018
gi|472242205	aspartate aminotransferase protein	385.8	26.1	1.837	0.048
gi|347841990	tryptophan synthase	611.0	28.3	1.542	0.024
gi|347832506	threonine synthase	348.4	16.4	1.560	0.047
gi|154692095	cysteine synthase	292.8	25.0	1.589	0.028
gi|347833148	glutamine synthetase	484.0	26.9	1.778	0.015
gi|347839014	histidine biosynthesis protein	184.6	9.3	1.840	0.027
gi|347828253	dihydrodipicolinate synthetase family protein	518.7	28.0	1.869	0.013
gi|347836881	D-3-phosphoglycerate dehydrogenase	656.4	25.5	1.758	0.018
gi|472242394	saccharopine dehydrogenase protein	338.7	36.2	1.743	0.039
gi|347441047	glycine dehydrogenase	286.9	12.3	1.708	0.029
gi|507414630	C-1-tetrahydrofolate synthase	905.4	31.2	1.737	0.031
gi|347831191	glutamate carboxypeptidase protein	298.0	23.2	1.977	0.020
gi|347841903	methionine aminopeptidase 1	221.2	20.3	2.040	0.021
gi|332313356	methionine aminopeptidase 2	73.1	10.3	2.044	0.027
gi|347829817	serine/threonine protein kinase	32.6	4.4	1.693	0.037
gi|472244536	glutamate-cysteine ligase protein	61.6	3.6	1.698	0.037
gi|347829487	5-methyltetrahydropteroyltriglutamate-homocysteine methyltransferase	2, 013.2	41.1	2.505	0.004
gi|347836712	glycine cleavage system H protein	116.0	22.0	1.982	0.025
gi|472236211	amino acid permease protein	39.7	4.0	1.999	0.031
gi|347830997	peptide methionine sulfoxide reductase	82.8	20.5	1.919	0.029
gi|472243795	aromatic-l-amino-acid decarboxylase protein	287.6	12.1	1.936	0.015
gi|347833024	lysine decarboxylase-like protein	79.7	8.6	1.585	0.033
gi|472246546	glutathione-dependent formaldehyde dehydrogenase	587.5	48.7	1.666	0.043
gi|347840830	NADH-cytochrome b5 reductase	305.5	23.0	1.545	0.029
gi|347827019	cytochrome P450 monooxygenase	31.4	2.4	1.722	0.042
gi|125949746	calcineurin	194.3	12.4	1.777	0.023
gi|154289817	chitin synthase	129.2	4.7	1.555	0.023
gi|347840218	sorbitol dehydrogenase	28.3	2.9	1.706	0.028
gi|347440923	aldehyde dehydrogenase	1, 070.9	48.0	1.865	0.021
gi|347833737	mitochondrial peroxiredoxin Prx1	42.8	7.6	1.856	0.044
gi|347828993	antioxidant	129.5	33.1	2.127	0.028
gi|347839043	superoxide dismutase	163.1	17.0	1.717	0.012
gi|166408944	flavohemoglobin	294.7	35.7	1.994	0.009
gi|347828340	oxidoreductase	305.1	14.93	2.119	0.045
gi|347841065	nuclear control of ATPase protein	84.7	4.7	0.219	0.001
gi|347836808	heat shock protein 70	3, 060.8	53.2	1.750	0.014
gi|472242753	30 kda heat shock protein	296.7	47.5	1.959	0.019
gi|347827157	heat shock protein 90	1, 603.7	37.7	1.650	0.032
gi|347830903	heat shock protein STI1	689.1	35.8	2.451	0.011
gi|347830415	heat shock protein Hsp88	1, 199.5	34.3	1.817	0.020
gi|347833633	heat shock protein	748.3	34.3	1.999	0.020
gi|154288804	short chain dehydrogenase	105.9	20.7	2.142	0.005
gi|347840162	translation initiation factor 3	284.6	46.8	1.905	0.031
gi|472245156	eukaryotic translation initiation factor 3 subunit	749.4	18.9	1.890	0.015
gi|229463757	eukaryotic translation initiation factor 3 subunit H	195.8	20.7	1.851	0.013
gi|229501208	eukaryotic translation initiation factor 3 subunit K	232.7	33.5	1.751	0.044
gi|347841080	eukaryotic translation initiation factor 2 subunit alpha	193.5	17.1	1.574	0.030
gi|347830243	eukaryotic translation initiation factor 4e	151.7	12.0	1.798	0.044
gi|347840917	actin-depolymerizing factor 1	519.9	53.6	1.959	0.018
gi|3182891	actin	1, 055.4	52.8	1.555	0.035
gi|347831507	actin binding protein	276.9	16.6	1.942	0.003
gi|347840551	actin related protein 2/3 complex	217.4	21.9	1.835	0.013
gi|347838304	F-actin capping protein beta subunit isoforms 1 and 2	156.0	27.7	1.595	0.044
gi|205716451	actin cytoskeleton-regulatory complex protein end 3	109.4	10.4	1.827	0.022
gi|347827283	actin lateral binding protein	691.2	50.3	2.621	0.002
gi|347441258	myosin regulatory light chain cdc4	327.6	43.9	1.775	0.049
gi|347838471	survival factor 1	321.9	28.4	1.608	0.038
gi|347441690	transcription factor HMG	78.8	21.8	3.565	0.004
gi|347838526	transcription factor CCAAT	39.1	3.5	4.970	0.001
gi|374093884	transcription regulator PAC1, partial	42.1	3.2	2.501	0.023
gi|472235708	cp2 transcription factor protein	92.2	6.2	1.748	0.040
gi|347826783	transcription initiation factor subunit	28.9	7.4	2.083	0.024
gi|347837746	transcription factor CBF/NF-Y	46.1	6.1	1.869	0.021
gi|347840266	transcription factor Zn, C_2_H_2_	50.5	1.7	3.407	0.003
gi|347837101	EF-hand calcium-binding domain protein	42.8	3.7	0.031	0.001
gi|472246130	cell division control protein cdc48 protein	1, 298.4	40.7	1.654	0.021
gi|472235945	cell lysis protein	103.7	20.5	1.930	0.025
gi|206558271	cell division cycle protein 123	38.6	3.9	1.809	0.050
gi|347828695	apoptosis-inducing factor 3	267.7	17.2	2.290	0.003
gi|472242094	thioredoxin protein	388.6	51.4	2.634	0.003
gi|472244889	sulfate adenylyltransferase protein	328.9	25.8	1.858	0.011
gi|347839319	protein disulfide-isomerase	542.3	39.1	1.862	0.031
gi|347442007	transaldolase	1, 216.6	50.2	1.984	0.022
gi|154703303	elongation factor 1-alpha	2, 637.4	50.0	1.831	0.034
gi|347830450	elongation factor 2	1, 896.6	44.6	1.688	0.020
gi|472244387	elongation factor 1-beta protein	597.2	40.0	2.006	0.024
gi|347841449	NAD-dependent formate dehydrogenase	1, 663.0	50.1	1.931	0.042
gi|347835785	26S protease regulatory subunit 6A	355.1	27.6	1.848	0.017
gi|472242788	proteasome component pre3 protein	101.7	23.9	1.942	0.023
gi|347841691	arp2/3 complex subunit Arc16	249.2	41.7	1.729	0.020
gi|154319207	26S protease regulatory subunit 7	221.9	19.4	2.009	0.026
gi|347833025	proteasome subunit alpha type 1	133.2	16.9	1.706	0.025
gi|347441407	protein kinase C substrate	282.5	18.1	1.703	0.028
gi|347827686	sec14 cytosolic factor	240.1	41.4	1.711	0.030
gi|347840528	peptidyl-prolyl cis-trans isomerase D	431.3	39.9	2.070	0.019
gi|563298153	inorganic pyrophosphatase	317.8	29.7	1.714	0.015
gi|347830035	aldose 1-epimerase	338.4	29.6	2.114	0.040
gi|347831189	carbohydrate-Binding Module family 48 protein	330.4	27.1	3.744	0.014
gi|347839149	carbohydrate-Binding Module family 50 protein	196.5	25.3	2.276	0.047
gi|347841295	cystathionine beta-synthase	416.0	26.0	1.790	0.031
gi|347842143	diphosphomevalonate decarboxylase	303.6	25.9	1.788	0.022
gi|347836348	protein phosphatase PP2A regulatory subunit A	414.1	21.1	1.576	0.045
gi|347838932	class I/II aminotransferase	340.3	23.9	1.844	0.015
gi|347831623	amidophosphoribosyltransferase	1, 467.6	20.8	1.573	0.025
gi|472236449	enoyl- hydratase isomerase protein	101.1	19.1	1.849	0.026
gi|472237246	tubulin-specific chaperone c protein	222.7	20.7	1.621	0.044
gi|347826898	trans-2-enoyl-CoA reductase	31.9	1.9	0.031	0.001
gi|347837864	1,3,8-naphthalenetriol reductase	89.0	19.6	2.213	0.029
gi|472243905	casein kinase i protein	148.3	19.8	1.591	0.043
gi|347831955	acetate kinase	193.1	18.9	1.726	0.015
gi|347839614	aspartyl aminopeptidase	293.3	18.8	1.564	0.036
gi|472238538	3-hydroxybutyryl-dehydrogenase protein	133.3	17.2	1.645	0.025
gi|347441025	arf gtpase-activating protein	249.9	17.2	2.074	0.008
gi|347828551	phosphatidyl synthase	72.6	9.4	1.967	0.029
gi|154294387	mitogen-activated protein kinase	101.9	17.1	1.664	0.039
gi|472240101	alpha beta hydrolase fold-3 domain protein	45.3	9.0	1.812	0.020
gi|347827703	BAR domain protein	271.6	43.4	1.751	0.037
gi|347830570	ThiJ/PfpI family protein	645.5	37.0	1.703	0.016
gi|347832713	DUF1688 domain-containing protein	437.7	27.6	1.726	0.034
gi|472245392	DUF718 domain-containing protein	75.6	27.3	1.803	0.019
gi|347836108	C2 domain-containing protein	286.0	23.6	1.947	0.021
gi|347833490	DUF757 domain-containing protein	74.8	22.4	1.840	0.045
gi|472245612	c6 finger domain protein	248.4	22.4	1.782	0.029
gi|347838618	UBX domain-containing protein	101.1	16.2	2.252	0.036
gi|472236354	yip1 domain-containing protein	66.0	11.1	2.052	0.033
gi|347836200	FAD binding domain-containing protein	117.4	10.7	2.072	0.015
gi|347836441	DUF89 domain-containing protein	69.4	6.0	1.638	0.027
gi|472240877	bar domain-containing protein	69.2	5.9	1.784	0.040
gi|347832303	acyl-CoA dehydrogenase domain protein	202.2	19.9	2.010	0.042
gi|472237107	saff domain-containing protein	94.8	8.5	1.933	0.015
gi|347828586	CUE domain-containing protein	53.8	3.1	3.833	0.008
gi|472244807	calponin domain protein	79.3	2.9	2.067	0.033
gi|563296966	KH domain protein	31.2	1.7	1.900	0.011
gi|347829378	R_3_H domain-containing protein	32.3	1.6	1.938	0.001
gi|347836748	pumilio domain-containing protein	37.9	1.4	2.313	0.007
gi|347836261	methyltransferase domain-containing protein	27.9	2.9	0.031	0.001
gi|154691472	eukaryotic peptide chain release factor subunit 1	426.9	30.8	1.912	0.036
gi|347837479	glia maturation factor gamma	102.7	30.6	1.703	0.028
gi|347837628	CORD and CS domain-containing protein	134.3	29.8	1.787	0.013
gi|347828828	ruvB-like helicase 1	417.5	30.4	1.502	0.035
gi|347442085	CND8	99.4	6.3	0.405	0.001
gi|156051430	40S ribosomal protein S3	1, 591.3	60.8	1.638	0.040
gi|347827805	40S ribosomal protein S5	418.3	38.5	1.531	0.044
gi|347835120	40S ribosomal protein S6	332.8	34.3	1.763	0.046
gi|347836429	40S ribosomal protein S7	276.1	30.4	1.857	0.007
gi|156043471	40S ribosomal protein S8	688.8	40.2	1.584	0.026
gi|154291145	40S ribosomal protein S10	106.2	25.4	1.891	0.016
gi|156061679	40S ribosomal protein S13	404.4	33.8	1.867	0.035
gi|472237384	40S ribosomal protein S18	546.8	42.3	1.902	0.018
gi|347837250	40S ribosomal protein S19	363.8	51.0	2.715	0.025
gi|347441467	40S ribosomal protein S21	157.1	63.6	2.762	0.018
gi|347829326	40S ribosomal protein S23	190.6	20.0	1.898	0.048
gi|156065881	40S ribosomal protein S24	348.1	32.6	1.861	0.040
gi|156065633	40S ribosomal protein S25	174.3	26.8	2.073	0.037
gi|347832333	40S ribosomal protein S27	322.4	37.8	1.823	0.028
gi|347828118	40S ribosomal protein S29	126.9	42.9	2.508	0.013
gi|347827513	40S ribosomal protein S30	63.1	16.1	0.199	0.002
gi|347828771	60S ribosomal protein L44	97.8	13.2	2.919	0.014
gi|156062084	60S ribosomal protein L9	1, 053.6	63.4	1.571	0.031
gi|229891536	54S ribosomal protein L4, mitochondrial	54.3	6.8	0.375	0.024
gi|156037530	60S ribosomal protein L12	608.9	40.0	1.562	0.010
gi|347832401	60S ribosomal protein L13	444.7	33.0	1.662	0.032
gi|347835805	60S ribosomal protein L6	611.8	33.0	1.670	0.023
gi|347836248	60S ribosomal protein L10	126.5	11.3	2.336	0.030
gi|347839766	60S ribosomal protein L16	271.7	29.7	2.055	0.039
gi|154316257	60S ribosomal protein L17	563.9	30.5	2.136	0.011
gi|154310248	60S ribosomal protein L19	409.8	29.4	2.652	0.009
gi|347840178	60S ribosomal protein L21	247.9	35.6	1.977	0.029
gi|347830985	60S ribosomal protein L23	425.6	48.9	1.936	0.030
gi|347835534	60S ribosomal protein L24	274.0	29.0	2.291	0.015
gi|347831348	60S ribosomal protein L26	236.5	36.8	2.174	0.030
gi|347441549	60S ribosomal protein L27a	708.8	48.3	1.603	0.018
gi|347841474	60S ribosomal protein L28	236.9	52.7	3.593	0.010
gi|472245831	60S ribosomal protein L31	295.4	48.0	2.230	0.019
gi|347826648	60S ribosomal protein L33	274.2	37.6	1.909	0.034
gi|154315039	60S ribosomal protein L35	140.2	18.9	2.744	0.024
gi|156036474	60S ribosomal protein L36	166.0	35.9	1.648	0.038
gi|154297648	60S acidic ribosomal protein P0	1, 277.7	41.7	1.896	0.029
gi|347835237	60S acidic ribosomal protein P1	553.2	41.2	2.379	0.011
gi|347838558	60S acidic ribosomal protein P2	500.4	55.9	2.178	0.012
gi|347441053	ribosome associated DnaJ chaperone Zuotin	635.2	25.3	1.863	0.029
gi|156044830	ribosome biogenesis protein Nhp2	106.9	9.8	1.594	0.024
gi|229485392	ribosome biogenesis protein erb1	56.1	4.2	1.636	0.045
gi|347837666	nuclear transport factor 2	236.2	28.2	2.249	0.020
gi|472246396	nuclear segregation protein	466.5	27.0	3.04	0.013
gi|347835094	leucyl-tRNA synthetase	722.1	25.9	1.809	0.016
gi|347835240	methionyl-tRNA synthetase	183.7	18.9	1.931	0.029
gi|347828755	tryptophanyl-tRNA synthetase	283.9	23.4	1.864	0.037
gi|563295297	histidyl-tRNA synthetase	286.9	21.9	1.691	0.027
gi|347835339	glutamyl-tRNA synthetase	353.0	21.5	1.783	0.032
gi|347841257	threonyl-tRNA synthetase	522.0	18.2	1.918	0.013
gi|347840344	valyl-trna synthetase	535.3	13.7	1.681	0.046
gi|347833265	aspartyl-tRNA synthetase	271.9	15.1	1.861	0.017
gi|347836347	phenylalanyl-tRNA synthetase beta chain	159.9	13.5	2.148	0.003
gi|347842507	tRNA methyltransferase	31.7	2.9	1.735	0.006
gi|347837080	polyadenylate-binding protein	621.1	19.8	1.755	0.039
gi|563292520	histone H1-binding protein	84.1	7.0	1.894	0.025
gi|472237673	oxysterol-binding protein	154.5	6.5	3.378	0.014
gi|154692219	glycogen synthase	204.9	11.1	1.884	0.038
gi|154308576	glucose-6-phosphate 1-dehydrogenase	365.5	25.1	1.986	0.023
gi|347833053	1,3-beta-glucan biosynthesis protein	131.7	10.6	2.131	0.033
gi|347841047	plasma membrane stress response protein	34.6	2.0	3.195	0.009
gi|347830640	methylenetetrahydrofolate reductase	196.2	13.4	1.552	0.019
gi|154309515	ca/CaM-dependent kinase-1	141.7	18.4	1.566	0.036
gi|347829911	GTP-binding nuclear protein Ran	301.8	38.1	1.732	0.025
gi|472236275	tRNA splicing endonuclease subunit protein	96.8	14.5	2.013	0.007
gi|347831289	RNA binding effector protein Scp160	853.4	22.1	1.568	0.050
gi|347839263	DNA-directed RNA polymerase I subunit	49.6	14.1	2.662	0.041
gi|347441996	HAD superfamily hydrolase	203.1	32.5	1.599	0.041
gi|347840552	ubiquitin carboxyl-terminal hydrolase	362.9	27.1	1.976	0.026
gi|347837756	ubiquitin-like protein SMT3	34.9	18.8	2.301	0.030
gi|472238757	ubiquitin-activating enzyme e1 1 protein	489.3	17.3	1.665	0.016
gi|154695558	ubiquitin-conjugating enzyme E2	36.3	7.5	1.579	0.042
gi|472241717	ubiquitin thioesterase protein	56.4	8.3	1.749	0.027
gi|347440894	translocon beta subunit Sbh1	225.3	44.6	1.753	0.042
gi|472236180	minor allergen alt a 7 protein	282.3	47.8	2.844	0.005
gi|472235513	anthranilate synthase component 2 protein	392.7	20.7	1.590	0.029
gi|347833273	nipsnap family protein	154.3	19.9	1.633	0.026
gi|347832071	phosphoglucomutase	1, 936.2	53.1	1.896	0.017
gi|347829895	phosphomannomutase	182.7	21.5	1.854	0.028
gi|347832016	N-acetylglucosamine-phosphate mutase	436.9	26.4	1.853	0.011
gi|347841616	UDP-galactopyranose mutase	549.0	33.1	2.149	0.020
gi|347841593	UDP-N-acetylglucosamine pyrophosphorylase	519.9	35.0	1.922	0.008
gi|472237006	UDP-glucose 4-epimerase gal10 protein	191.1	20.5	1.867	0.009
gi|347441001	mannose-1-phosphate guanyltransferase alpha-a	584.1	36.3	1.631	0.033
gi|472241485	nad h-dependent d-xylose reductase xyl1 protein	247.9	28.6	1.541	0.046
gi|347828612	transketolase	1, 284.8	41.2	2.020	0.013
gi|154321267	phosphoketolase	883.5	24.4	1.836	0.042
gi|347842358	acetyl-CoA acetyltransferase	449.4	46.3	1.982	0.018
gi|347830285	phospho-2-dehydro-3-deoxyheptonate aldolase	460.2	36.1	1.950	0.027
gi|347840715	3-isopropylmalate dehydratase	593.0	29.8	1.519	0.019
gi|347440697	cyanide hydratase/nitrilase	353.7	17.0	2.551	0.012
gi|347832595	aldo/keto reductase family oxidoreductase	497.6	42.5	1.999	0.018
gi|154322845	aldo/keto reductase	327.8	28.9	1.724	0.044
gi|347838695	nitroreductase family protein	228.3	32.7	1.893	0.018
gi|154293270	glucose 1-dehydrogenase	263.4	27.8	1.636	0.043

a *Fold: the average ratio (control/TTO-treated) of protein levels from three biological replicates as determined by iTRAQ approach. A protein was considered a differential expression protein as it exhibited a >1.5-fold or < 0.67-fold change and P < 0.05*.

### GO analysis of DEPs

**Figure 2:**
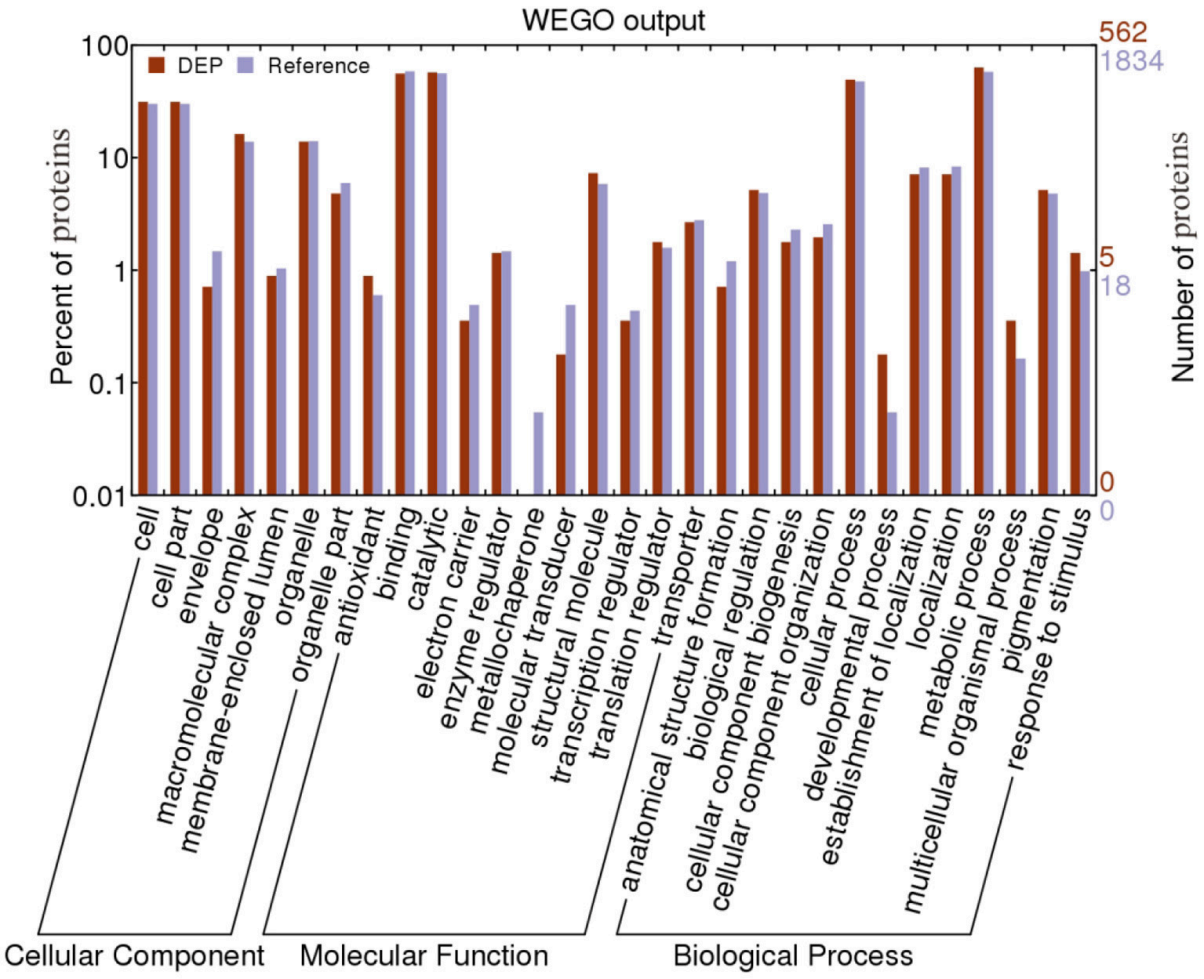
Gene Ontology (GO) analysis of differentially expressed proteins (DEPs) identified in *B. cinerea* cells treated with TTO.

GO analysis was conducted to identify significantly enriched GO functional groups. DEPs were categorized by biological process, cellular component, and molecular function. Of the 718 DEPs, 562 were annotated and classified into 30 functional groups (Figure 2). Biological processes accounted for 12 GO terms (with “metabolic process” accounting for 44.11% of these, and “cellular process” 34.32%). Cellular components accounted for 7 GO terms, dominated by “cell” (31.60%) and “cell part” (31.60%). Molecular functions accounted for 11 GO terms, the most abundant being “catalytic” (44.72%) and “binding” (43.61%).

The agriGO analysis tool was used to detect and visualize significantly enriched GO terms associated with the 562 annotated proteins, with an adjusted *p*-value cutoff of 0.05. Significant functions included “regulation of biological quality” (GO:0065008, *p* = 0.033) and “primary metabolic process” (GO:0044238, *p* = 0.016). There are 5 DEPs, accounting for about 45.45% of the total protein in regulation of biological quality. And 189 DEPs, accounting for about 73.82% of the total protein in primary metabolic process.

### KEGG analysis of DEPs

Proteins typically do not exercise their functions independently, but coordinate with each other to complete a series of biochemical reactions. Pathway analysis can help reveal cellular processes involved in disease mechanisms or drug action. Using the KEGG database as a reference, 562 DEPs were linked to 133 different pathways. Glycolysis, the TCA cycle, and purine metabolism were among the pathways most significantly altered by exposure to TTO.

### Confocal microscopy

**Figure 3:**
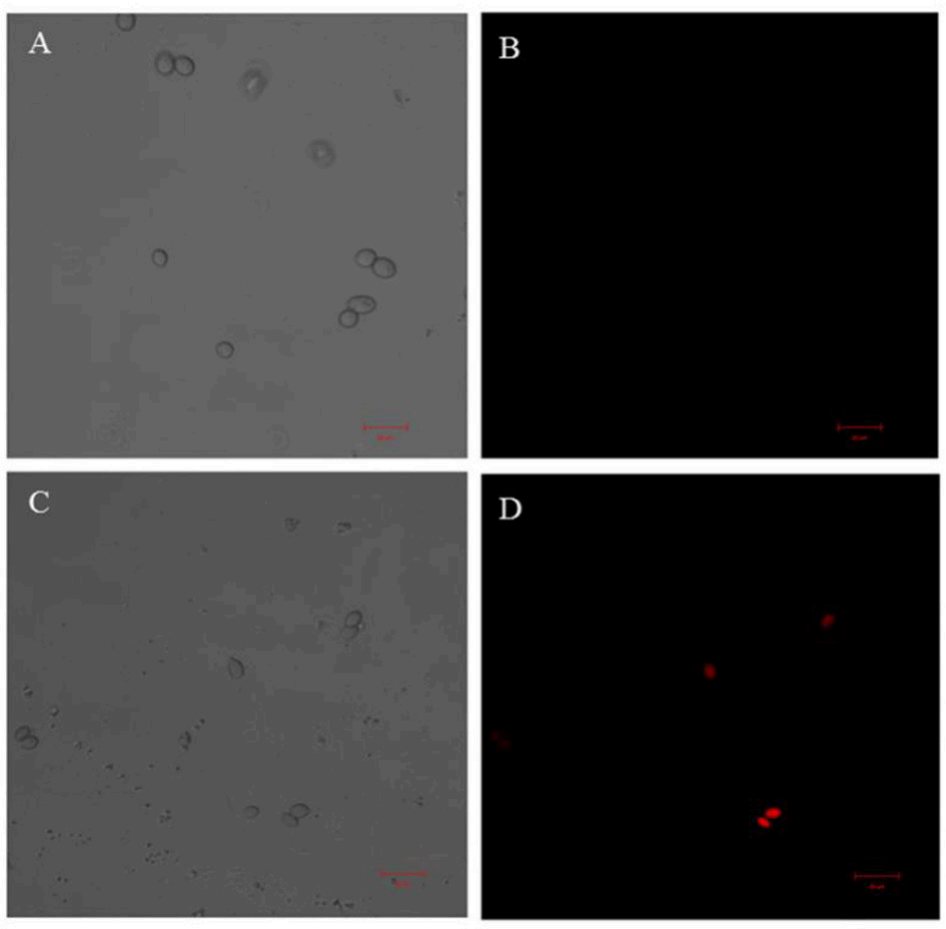
Effect of TTO treatment on cytoplasmic membranes in *B. cinerea* cells. Images were acquired by confocal microscopy using the fluorescent indicator PI. *B. cinerea* spores were incubated without TTO **(A,B)**, or with 5 mL/L TTO **(C,D)**. Bright-field **(A,C)** and fluorescent **(B,D)** images are shown. Red fluorescence indicates PI staining of nucleic acids. Scale bar: 20 μm.

Confocal laser scanning microscopy was used to investigate *B. cinerea* cell membrane integrity after TTO treatment. PI easily penetrates a membrane-damaged cell and binds to DNA, resulting in red fluorescence. *B. cinerea* cells were examined by both bright-field microscopy (Figures 3A,C) and fluorescence microscopy (Figures 3B,D). Control cells have no detectable red fluorescence (Figure 3B), indicating that they have intact cell membranes. In contrast, red fluorescence was observed after cells were treated for 2 h with TTO at 5 mL/L (Figure 3D). These results suggest that TTO compromises the integrity of the *B. cinerea* cell membrane, potentially causing cell death.

### Enzyme activities related to TCA cycle

**Figure 4:**
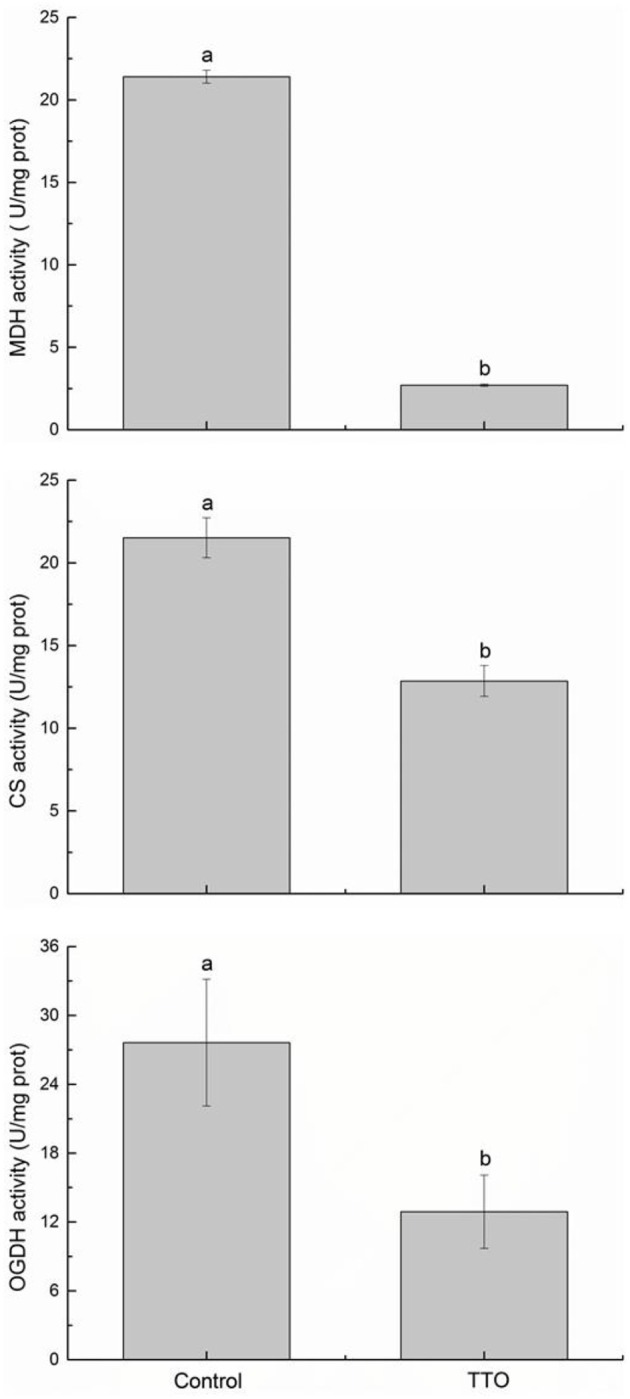
Effect of TTO treatment on MDH, CS, and OGDH activities in *B. cinerea*. Vertical bars represent the standard deviation of the means. a,b: significant differences at *P* < 0.05 level based on Duncan's multiple range tests.

Because the iTRAQ analysis clearly implicated the TCA cycle as a possible TTO target, we investigated the activities of MDH, CS, and OGDH, three key enzymes related to the TCA cycle (Figure 4). The results indicate that activities for these enzymes decreased significantly in TTO-treated cells (87.4, 53.3, and 40.3%, respectively), consistent with our observation that the MDH, CS, and OGDH proteins are significantly down-regulated in TTO-treated cells.

## Discussion

The antifungal activity of essential oils is probably based on their ability to significantly reduce total lipid and ergosterol content, thereby disrupting membrane permeability and resulting in leakage of cell components such as ATP, DNA, and potassium ions (Tian et al., 2011; Tao et al., 2014; Cui et al., 2015). Our previous study demonstrated that TTO considerably increases membrane permeability, causing extrusion of abundant material (Shao et al., 2013b; Yu et al., 2015) and decreasing intracellular ATP in *B. cinerea* (Li et al., 2017b). In this study, observations using confocal laser scanning microscopy indicate that TTO damages the *B. cinerea* cell membrane, potentially causing the release of internal material such as ATP.

Levels for many DEPs related to glycolysis metabolism, such as glucose-6-phosphate isomerase, 6-phosphofructokinase, phosphoenolpyruvate carboxykinase, fructose-1, 6-bisphosphatase, and enolase, are decreased by TTO treatment (Table 1). Glucose-6-phosphate isomerase catalyzes the conversion of glucose-6-phosphate into fructose 6-phosphate in the second step of glycolysis (Achari et al., 1981). 6-phosphofructokinase is a key enzyme in the control of the glycolytic pathway in nearly all cells (Wang et al., 2016). The activity of this enzyme is controlled by several metabolites, most notably its two substrates, fructose 6-phosphate and ATP. Glycolysis is also an important pathway for energy production in the cytosol of plant cells. Our results suggest that TTO inhibits glycolysis and may affect energy supply in *B. cinerea*.

Mitochondria are the primary sites of aerobic respiration in eukaryotic cells. They generate energy for cellular functions through oxidative phosphorylation and the TCA cycle, and also play a crucial role in regulating the apoptosis (Shaughnessy et al., 2014). In this study, several proteins associated with the mitochondrial respiratory chain and TCA cycle, such as ATP synthase D chain, ATP synthase subunit e, MDH, CS, and OGDH, were significantly down-regulated in cells treated with TTO (Table 1). ATP synthase D chain and ATP synthase subunit e are involved in the biosynthesis of ATP. Dill oil inhibits mitochondrial ATPase activity and dehydrogenase activities, and affects mitochondrial function in *Aspergillus flavus* (Tian et al., 2012). Mustard essential oils decrease intracellular ATP and increase extracellular ATP in *E. coli* O157:H7 and *Salmonella typhi* (Turgis et al., 2009). Citral decreases intracellular ATP content, increases extracellular ATP content, inhibits the TCA pathway, and decreases the activities of CS and α-ketoglutarate dehydrogenase in *Penicillium digitatum* (Zheng et al., 2015). Our additional study demonstrates that TTO treatment significantly inhibits the activities of MDH, CS, and OGDH (Figure 4). In our previous study, we found that TTO decreases intracellular ATP and the activities of MDH, succinate dehydrogenase, ATPase, CS, isocitrate dehydrogenase, and α-ketoglutarate dehydrogenase, disrupting the TCA cycle in *B. cinerea* (Li et al., 2017b). The down-regulation of two MDHs suggests that the Krebs cycle is not completely functional in *Paracoccidioides lutzii* upon exposure to argentilactone (Prado et al., 2014). Together, these results imply that TTO affects proteins in *B. cinerea* involved in glycolysis, the TCA cycle, and ATP synthesis, thereby disrupting the TCA cycle, interrupting energy metabolism, and inducing mitochondrial dysfunction.

Cytochrome c (cyt c) is a hemoglobin located in the inner mitochondrial membrane, and is responsible for transferring electrons between mitochondrial electron transport chain complexes III and IV (Reed, 1997; Lo et al., 2017). ATP is produced by the aerobic mitochondrial respiratory chain. Abnormal cyt c disrupts the mitochondrial respiratory chain and impacts ATP production (Zhou et al., 2015). Our study shows that cyt c is up-regulated in *B. cinerea* after TTO treatment at 5 mL/L (Table 1). The increase in cyt c levels may improve the performance of the oxidative respiratory chain, perhaps as a protective response to TTO toxicity.

Purines are one of the building blocks for nucleic acids. Their synthesis pathways generate many kinds of energy molecules (Qian et al., 2014). Inosine 5′-monophosphate dehydrogenase (IMPDH) is a rate-controlling enzyme in the *de novo* synthesis of the guanine nucleotide, and plays crucial roles in cell growth and proliferation (Fotie, 2016). IMPDH inhibition reduces guanine nucleotide pools and interrupts cellular functions such as DNA replication, RNA synthesis, and signal transduction (Weber, 1983; Weber et al., 1996). These effects are associated with cell cycle disruption, cellular differentiation, and apoptosis (Vitale et al., 1997; Yalowitz and Jayaram, 2000). Nucleoside diphosphate kinases (NDPK) are critical enzymes related to the maintenance of intracellular nucleotide levels, and catalyze the conversion of nucleoside triphosphates to nucleoside diphosphates in all living organisms (Véron et al., 1994). Both NDPK and AK can mediate the conversion of adenosine into ATP by ADP and AMP (Senft and Crabtree, 1983). In our study, TTO treatment decreased IMPDH levels (Table 1). Furthermore, levels of adenosine kinase AK and NDPK were also reduced after TTO treatment (Table 1). From these results, we can conclude that TTO may block the accumulation of energy and disrupt the cell cycle, ultimately inducing apoptosis.

## Conclusion

**Figure 5:**
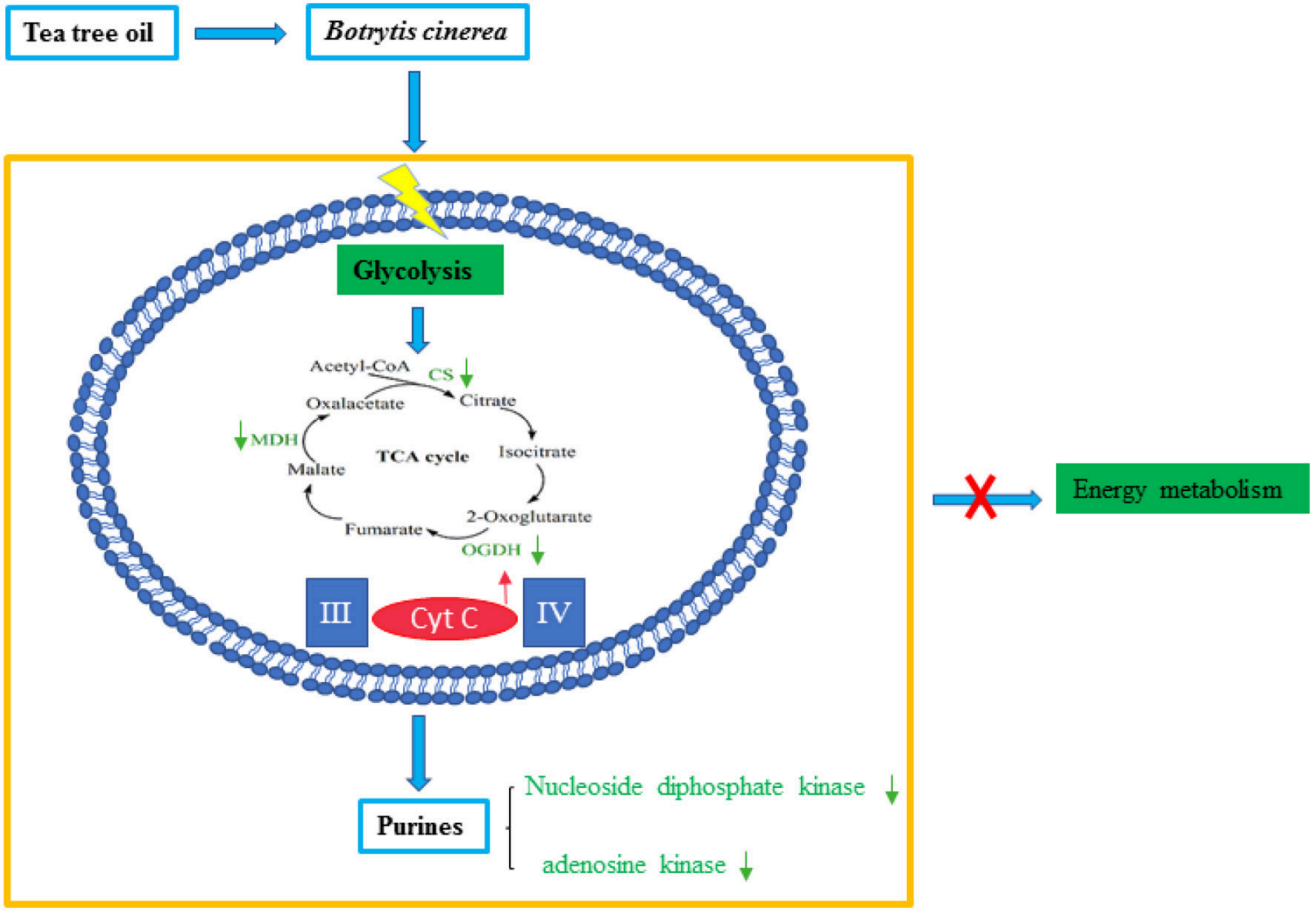
Model summarizing antifungal effects of TTO in *B. cinerea*. Green arrows indicate down-regulation and red arrows indicate up-regulation.

The effect of TTO treatment on proteins in *B. cinerea* is summarized in Figure 5. We found that important metabolic pathways, including glycolysis, the TCA cycle, and purine metabolism, were compromised by TTO treatment, while cyt c increased. We conclude that the disruption of energy metabolism by TTO contributes to its antifungal activity against *B. cinerea*.

## Author contributions

JX and XS designed the experiments. JX and YW performed the experiments. FX and HW analyzed the data. JX, XS, and HW drafted the manuscript. All authors read and approved the final manuscript.

### Conflict of interest statement

The authors declare that the research was conducted in the absence of any commercial or financial relationships that could be construed as a potential conflict of interest.
